# Changing Complication Profiles in the Era of Robotic Ivor Lewis Esophagectomy: A Comparative Analysis of Open, Hybrid, and Fully Robotic Techniques

**DOI:** 10.3390/cancers18060954

**Published:** 2026-03-15

**Authors:** Sebastian Weberskirch, Neele Wilkens, Ann-Kathrin Eichelmann, Jennifer Merten, Nader El-Sourani, Mazen A. Juratli, Andreas Pascher, Jens Peter Hoelzen

**Affiliations:** Department of General, Visceral and Transplant Surgery, University Hospital Muenster, University of Muenster, Albert-Schweitzer-Campus 1, 48149 Muenster, Germany; sebastian.weberskirch@ukmuenster.de (S.W.); neele.wilkens@ukmuenster.de (N.W.); ann-kathrin.eichelmann@ukmuenster.de (A.-K.E.); jennifer.merten@ukmuenster.de (J.M.); nader.el-sourani@ukmuenster.de (N.E.-S.); mazen.juratli@ukmuenster.de (M.A.J.); andreas.pascher@ukmuenster.de (A.P.)

**Keywords:** esophageal cancer, robotic esophagectomy, RAMIE, Ivor Lewis, chylothorax, paraconduit herniation, neo-esophagus-airway fistula, minimally invasive surgery, lymphadenectomy

## Abstract

**Background:** Novel robotic surgical techniques have substantially improved the safety and outcomes of Ivor Lewis esophagectomy, offering greater precision, reduced surgical trauma, and more radical lymphadenectomy compared to conventional approaches. While perioperative results are increasingly promising, the adoption of robotic technology appears to be accompanied by an emerging set of procedure-specific complications not previously encountered—or encountered with different frequency—in open surgery. Understanding this evolving complication profile is essential to fully realize the oncological potential of robotic esophagectomy. **Methods:** This retrospective single-center study compared 407 consecutive patients undergoing Ivor Lewis esophagectomy at a high-volume center (OPE *n* = 163; HRB *n* = 75; FRB *n* = 169; 2012–2023) regarding three pathophysiologically motivated primary endpoints within 12 months: paraconduit herniation, chylothorax, and neo-esophagus–airway fistula. **Results:** One-year survival was 71.8%, 74.7%, and 82.2% (*p* = 0.073). Chylothorax was significantly more frequent in FRB (12.4%) than in OPE (2.5%) or HRB (2.7%) (*p* < 0.001), with surgical approach as sole independent predictor. Lymphangiography in three FRB patients demonstrated thoracic duct integrity in all; leakage originated from the celiac lymphadenectomy field ascending transhiatally—suggesting a distinct mechanism potentially linked to surgical radicality. All cases resolved conservatively. Neo-esophagus–airway fistula occured significantly less frequently with robotic approaches (FRB 0.6% vs. OPE 4.9%; *p* = 0.031). Paraconduit herniation did not differ significantly within 12 months (*p* = 0.272). **Conclusions:** The complication profile of robotic Ivor Lewis esophagectomy reflects its oncological ambition: elevated chylothorax rates may correlate with radical lymphadenectomy and represent an acceptable trade-off within a multimodal treatment strategy. Fistula risk is meaningfully reduced. These findings support robotic esophagectomy as a safe and effective approach in experienced centers.

## Simple Summary

Robot-assisted minimally invasive esophagectomy (RAMIE) is increasingly used in esophageal cancer surgery because it improves surgical precision and facilitates more extensive lymphadenectomy. However, robotic surgery may also be associated with a distinct complication profile. In this retrospective single-center study, we analyzed 407 consecutive patients undergoing Ivor Lewis esophagectomy using open, hybrid robotic, or fully robotic techniques. We focused on three clinically relevant complications: paraconduit herniation, chylothorax, and neo-esophagus–airway fistula. Fully robotic surgery was associated with a significantly higher chylothorax rate, whereas neo-esophagus–airway fistulas occurred significantly less often compared with open surgery. Lymphangiography in persistent chylothorax cases confirmed an intact thoracic duct, suggesting transhiatal lymphatic leakage from the abdominal lymphadenectomy field rather than classical thoracic duct injury. These findings highlight procedure-specific risks and may support improved preventive strategies and postoperative management in robotic esophagectomy.

## 1. Introduction

Esophageal cancer remains a highly lethal malignancy with persistently poor long-term outcomes. In 2022, approximately 511,000 new cases were diagnosed worldwide, accounting for about 2.6% of all malignant neoplasms and ranking it eleventh among all cancers globally [1]. Despite advances in multimodal treatment, population-level improvements have been modest: in Germany, esophageal cancer still accounts for 3.7% of cancer-related deaths in men and 1.3% in women [2], and age-standardized incidence and mortality rates have changed only marginally since 1999 [3]. Accordingly, relative five-year survival remains among the lowest across solid tumors, with reported rates as low as 25%, largely reflecting advanced stage at diagnosis in most patients [2].

At diagnosis, only 30–40% of patients present with potentially resectable disease. In the absence of distant metastases, and provided both operability and oncological resectability are confirmed, esophagectomy—typically incorporated within a multimodal treatment strategy with neoadjuvant chemotherapy or chemoradiotherapy—remains the cornerstone of curative treatment [3,4]. Nevertheless, even in experienced hands, esophagectomy is among the most demanding procedures in surgical oncology, with reported overall complication rates approaching 59% and 90-day mortality ranging from 4.5% to 13% [5,6,7].

The introduction of minimally invasive esophagectomy (MIE) represented a major advance, reducing surgical trauma and improving perioperative recovery compared with open surgery, with demonstrable benefits for short- and long-term quality of life [8,9,10]. Robotic-assisted minimally invasive esophagectomy (RAMIE) represents the next evolutionary step, specifically addressing key limitations of conventional thoracoscopic approaches—most notably restricted instrument articulation and limited maneuverability in narrow anatomical compartments [11,12]. With enhanced visualization and wristed instrumentation, RAMIE facilitates meticulous dissection in the upper mediastinum and along the recurrent laryngeal nerve and has been associated with improved textbook outcomes and potentially superior long-term survival [13,14,15].

Yet, the same technical advantages that enable more radical lymphadenectomy may also give rise to an evolving spectrum of rare but clinically meaningful complications.

Robot-assisted esophagectomy was introduced at the University Hospital of Münster (UKM) in late 2018, and full robotic RAMIE has been performed as the institutional standard since 2021. Although robotic surgery is widely perceived as a driver of improved perioperative outcomes, its technical characteristics may also shift the pattern of postoperative morbidity. In particular, paraconduit herniation has been reported more frequently after minimally invasive esophagectomy than after open procedures, a phenomenon commonly attributed to a wider hiatal opening and reduced postoperative adhesion formation [16,17,18]. Similarly, increased chylothorax rates after RAMIE have been described and are often linked to more extensive lymphadenectomy and meticulous dissection of lymphatic structures [19,20]. Finally, neo-esophagus–airway fistula represents a rare but devastating complication, potentially influenced by the extent of mediastinal dissection and tissue vulnerability following neoadjuvant treatment [21,22].

Although infrequent, these complications carry substantial clinical weight: they prolong recovery, delay oncological rehabilitation, and can decisively shape postoperative quality of life. Defining their true incidence and identifying modifiable risk factors therefore carries direct clinical relevance, informing operative strategy, perioperative surveillance, and patient counseling. This study systematically evaluates the incidence and characteristics of paraconduit herniation, chylothorax, and neo-esophagus–airway fistula within 12 months after Ivor Lewis esophagectomy in a consecutive cohort of 407 patients treated by open, hybrid robotic, or fully robotic approaches at a single high-volume center.

## 2. Materials and Methods

### 2.1. Study Design

This retrospective single-center study compared three surgical approaches to Ivor Lewis esophagectomy—open esophagectomy (OPE), hybrid robotic-assisted minimally invasive esophagectomy (HRB), and full robotic RAMIE (FRB)—with respect to three predefined primary endpoints: chylothorax, paraconduit herniation, and neo-esophagus–airway fistula occurring within 12 months after surgery. The three approaches were implemented sequentially at our institution over the study period, reflecting the stepwise transition from open surgery to hybrid and subsequently to full robotic techniques. The study period spanned January 2012 to December 2023 and was conducted at the University Hospital Münster (UKM), a high-volume academic tertiary referral center. Ethical approval was obtained from the Ethics Committee of the Medical Association of Westphalia-Lippe and the University of Münster (reference: 2022-123-f-S), and the study was performed in accordance with the Declaration of Helsinki.

### 2.2. Inclusion and Exclusion Criteria

All adult patients (≥18 years) undergoing Ivor Lewis esophagectomy at UKM during the study period were eligible if histology confirmed adenocarcinoma or squamous cell carcinoma of the thoracic esophagus or gastroesophageal junction and the tumor was considered surgically resectable either at initial staging or following neoadjuvant therapy. Group assignment reflected the stepwise institutional transition from open to hybrid robotic and subsequently fully robotic surgery, implemented as sequential epochs without individual case selection (OPE: January 2012–January 2019; HRB: December 2018–December 2020; FRB: January 2021 onward). The transition from hybrid to fully robotic surgery was enabled by the institutional upgrade from the da Vinci Si to the da Vinci Xi system, which for the first time allowed a fully robotic abdominal phase across all quadrants. Within each epoch, all consecutive eligible patients underwent the respective approach; no discretionary technique selection was applied. Patients undergoing cervical anastomosis (McKeown procedure) as well as those requiring intraoperative conversion or termination of the procedure were excluded.

### 2.3. Preoperative Workup and Multidisciplinary Planning

Preoperative staging followed a standardized institutional protocol including medical history, physical examination, upper endoscopy with biopsy, endoscopic ultrasound, and cross-sectional imaging (CT and PET/CT where indicated). Treatment strategy—including the indication for neoadjuvant therapy—was determined in a multidisciplinary tumor board in accordance with national guideline recommendations. Neoadjuvant treatment consisted of either FLOT chemotherapy (fluorouracil, leucovorin, oxaliplatin, docetaxel) or CROSS chemoradiotherapy (carboplatin/paclitaxel with concurrent radiotherapy), selected based on tumor stage and histology.

### 2.4. Surgical Procedures

All procedures followed a standardized two-phase Ivor Lewis esophagectomy technique, comprising an abdominal and thoracic phase with gastric conduit formation, D2 lymphadenectomy, and intrathoracic end-to-side esophagogastrostomy using a circular stapler. Anastomotic perfusion was routinely assessed intraoperatively using indocyanine green fluorescence imaging. The hiatal opening was not routinely narrowed by cruroplasty in any group. A detailed description of the operative technique has been published previously [23].

Open esophagectomy (OPE) was performed via upper midline laparotomy and right posterolateral thoracotomy. Lymphadenectomy in both phases was carried out using a vessel-sealing device (LigaSure). The thoracic duct was secured by mass ligature at the diaphragmatic hiatus without direct anatomical identification.

The hybrid approach (HRB) combined a laparoscopic abdominal phase with a robot-assisted thoracic phase with a small right-sided supply incision (~8 cm) for sample collection and anastomosis construction without formal thoracotomy. The thoracic phase was performed robotically using the da Vinci Si system (Intuitive Surgical, Sunnyvale, CA, USA), enabling selective identification and non-absorbable clip ligation of the thoracic duct under direct vision.

Full robotic RAMIE (FRB) was performed using the da Vinci Xi system (Intuitive Surgical, Sunnyvale, CA, USA) for both abdominal and thoracic phases, following standardized protocols including total mesoesophageal excision [23]. Abdominal lymphadenectomy was primarily performed using monopolar dissection, supplemented by vessel-sealing devices for larger lymphatic structures. Thoracic duct management was identical to HRB. In the FRB group, similar to the HRB group, a small right-sided supply incision (~8 cm) without formal thoracotomy was also performed.

Reconstruction was performed uniformly across all approaches. After transposition of the gastric conduit into the thoracic cavity, an intrathoracic end-to-side esophagogastrostomy was fashioned using a 29 mm powered circular stapler, with the anvil introduced directly through the thoracic access and secured in a purse-string suture at the proximal esophageal stump under direct vision, and the stapler introduced through a small gastrotomy at the conduit tip. In the HRB and FRB cohorts, ICG fluorescence angiography was routinely performed to assess blood flow in the gastric tube and guide the placement of the anastomosis [24]. All patients received prophylactic endoluminal vacuum therapy (EVT) postoperatively. Anastomotic integrity was assessed by routine endoscopy on postoperative day 5. In cases of endoscopic uncertainty, a contrast swallow study or contrast-enhanced CT was performed as complementary modalities [25,26,27,28].

### 2.5. Outcome Measures

Primary endpoints—assessed within 12 months postoperatively—were as follows: (1) paraconduit herniation, (2) chylothorax, and (3) neo-esophagus–airway fistula. Primary endpoint rates are reported for the entire cohort and additionally restricted to 12-month survivors to account for competing risk due to early mortality and to provide an appropriate denominator for late-onset complications such as paraconduit herniation. One-year overall survival was derived from the institutional master database (UKM Esophagectomy Registry 2012–2024, matched by OPS date).

Secondary outcomes comprised 30-day and in-hospital mortality, major complications (Clavien–Dindo grade ≥ IIIb), anastomotic leak, pneumonia, operative time, intraoperative blood loss, ICU length of stay, total postoperative hospital stay, and harvested lymph node count. Anastomotic leak was classified as a secondary endpoint because the anastomotic technique—intrathoracic end-to-side circular stapled esophagogastrostomy—was held constant across all three surgical approaches, precluding its use as a technique-discriminating primary endpoint.

### 2.6. Statistical Analysis

All statistical analyses were performed using R (version 4.4.1, R Foundation for Statistical Computing, Vienna, Austria). Continuous variables are presented as median (interquartile range, IQR); categorical variables as absolute numbers and percentages. Three-group comparisons employed the Kruskal–Wallis test for continuous variables and Pearson’s chi-square test for categorical variables. Logistic regression models were fitted using a binomial distribution with logit link function (BFGS optimization); odds ratios (OR) with 95% confidence intervals (CI) and Wald test *p*-values are reported. The FRB group served as the reference category throughout. Multivariable models were adjusted for age, sex, BMI, ASA classification, and neoadjuvant therapy type. For the fistula model, neoadjuvant therapy was incorporated as a binary variable (any vs. none) due to complete data separation, as no event occurred in patients without neoadjuvant therapy. A two-sided *p*-value < 0.05 was considered statistically significant. The study is reported in accordance with the STROBE statement for observational studies.

## 3. Results

### 3.1. Patient Characteristics and Baseline Data

Of all 495 patients assessed for eligibility during the study period, 88 were excluded (McKeown procedure, hybrid open procedure, intraoperative conversion or aborted procedure, neoadjuvant therapy other than radio-/radiochemotherapy), leaving 407 patients for final analysis. Baseline characteristics are summarized in Table 1. Patients in the FRB cohort were significantly older than those undergoing OPE or HRB (median 66.0 vs. 62.3 and 63.6 years, respectively; *p* = 0.012). Sex distribution and BMI were comparable between groups (*p* = 0.508 and *p* = 0.081, respectively). Comorbidity burden was higher in the robotic cohorts, reflected by an increased Charlson Comorbidity Index (median 5.0 vs. 4.0 in OPE; *p* < 0.001) and a higher prevalence of coronary artery disease (7.4% in OPE vs. 16.0% in HRB and 19.5% in FRB; *p* = 0.005). Adenocarcinoma was the predominant histology across all cohorts, with a significantly higher proportion in HRB and FRB compared to OPE (*p* < 0.001). Neoadjuvant therapy was administered more frequently in FRB than in OPE or HRB (91.7% vs. 79.1% and 80.0%; *p* = 0.003).

### 3.2. Primary Endpoints and One-Year Survival

**Figure 1:**
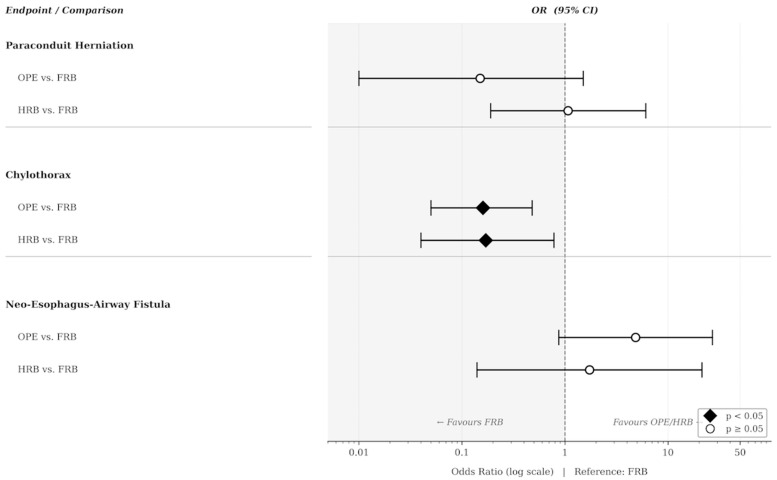
Forest Plot: Multivariable odds ratios (OR) with 95% confidence intervals (CI) for the three primary endpoints, with full robotic esophagectomy (FRB) as the reference group. OR < 1 indicates lower risk compared to FRB; OR > 1 indicates higher risk. Filled diamonds (◆) indicate statistically significant associations (*p* < 0.05); open circles (○) indicate non-significant results. Models were adjusted for age, sex, BMI, ASA classification, and neoadjuvant therapy type. For the fistula model, neoadjuvant therapy was recoded as binary (any vs. none) due to complete separation. OPE, open esophagectomy; HRB, hybrid robotic esophagectomy; FRB, full robotic esophagectomy; OR, odds ratio; CI, confidence interval.

One-year overall survival was 71.8% (117/163) after OPE, 74.7% (56/75) after HRB, and 82.2% (139/169) after FRB (*p* = 0.073). To account for differences in early mortality between cohorts, primary endpoint rates are reported for the overall cohort and additionally among 12-month survivors (Table 2, Figure 1).

#### 3.2.1. Paraconduit Herniation

Within 12 months, paraconduit herniation occurred in 1/163 OPE patients (0.6%), 2/75 HRB patients (2.7%), and 5/169 FRB patients (3.0%) (*p* = 0.272). Among 12-month survivors, rates were 0.9%, 3.6%, and 3.6%, respectively. Multivariable regression identified only increasing age as significant predictor (OR 0.93, 95% CI 0.88–0.99, *p* = 0.019), likely reflecting limited long-term survival in older patients (Table 3). Notably, 6 of 8 patients diagnosed with paraconduit herniation required surgical repair during the observation period. However, no herniation-related mortality was observed within the 12-month follow-up period.

#### 3.2.2. Chylothorax

Chylothorax occurred significantly more frequently in FRB (12.4%, 21/169) than in OPE (2.5%, 4/163) or HRB (2.7%, 2/75) (*p* < 0.001). Among 12-month survivors, chylothorax had occurred in 3.4% of OPE, 3.6% of HRB, and 11.5% of FRB patients—reported among survivors to account for competing risk of early mortality (Table 2). In multivariable analysis, surgical approach remained the only independent predictor of chylothorax (OPE vs. FRB: OR 0.16, 95% CI 0.05–0.48, *p* = 0.001; HRB vs. FRB: OR 0.17, 95% CI 0.04–0.78, *p* = 0.022) (Table 3). All 21 FRB-associated cases were managed conservatively. Radiographic lymphangiography was performed in three patients and demonstrated an intact thoracic duct in all cases. In these patients, lymphatic leakage originated from the celiac lymphadenectomy field and ascended transhiatally through a non-adherent hiatal gap into the thoracic cavity. Of the 27 patients who developed chylothorax, 5 (18.5%) died within 12 months from causes unrelated to the chylothorax itself.

#### 3.2.3. Neo-Esophagus–Airway Fistula

Neo-esophagus–airway fistula occurred in 8/163 OPE patients (4.9%), 1/75 HRB patients (1.3%), and 1/169 FRB patients (0.6%) (*p* = 0.031). All 10 fistula events occurred exclusively in patients who had received neoadjuvant therapy (CROSS protocol with concurrent radiotherapy). Notably, 7 of the 8 OPE patients with fistula died within 12 months, resulting in a markedly lower event rate when analyses were restricted to 12-month survivors (0.9%). Causes of death were predominantly related to sepsis and respiratory failure. In univariable regression, OPE was associated with an 8.67-fold-higher risk compared to FRB (OR 8.67, 95% CI 1.07–70.1, *p* = 0.043); this association was attenuated in the multivariable model (OR 4.84, *p* = 0.072), likely reflecting the low number of events (Table 3).

### 3.3. Secondary Endpoints

Secondary outcomes are summarized in Table 2. Anastomotic leak rates decreased stepwise from OPE (37.4%) to HRB (22.7%) and FRB (13.6%) (*p* < 0.001). Of note, the institutional protocol includes routine postoperative day-5 endoscopy with prophylactic EVT, which increases detection of minor anastomotic abnormalities compared with more restrictive diagnostic definitions. Pneumonia occurred significantly less frequently after FRB (13.0%) than after OPE (28.8%) or HRB (24.0%) (*p* = 0.002). Rates of major complications (Clavien–Dindo ≥ IIIb) were comparable between groups (OPE: 21.5%, HRB: 14.7%, FRB: 13.0%; *p* = 0.106), as were 30-day mortality (*p* = 0.502) and in-hospital mortality (*p* = 0.084). Compared with OPE, FRB was associated with markedly lower blood loss (median 80 vs. 400 mL), shorter ICU stay (median 2.0 vs. 10.0 days), shorter postoperative hospital stay (median 16.0 vs. 22.6 days), and a higher lymph node yield (median 32 vs. 25 nodes) (all *p* < 0.001).

## 4. Discussion

Robotic-assisted surgery is not an end in itself, but rather an enabling technology that allows highly complex oncological procedures to be performed in a minimally invasive setting, including in patients with substantial comorbidity who would previously have been considered at prohibitive operative risk. The advantages of the robotic platform are multifaceted: three-dimensional high-definition magnification and articulated instruments with enhanced degrees of freedom facilitate precise dissection and controlled tissue handling in anatomically confined spaces, thereby reducing blood loss and surgical trauma [29]. These technical properties translate into a reduced physiological burden of surgery and are associated with fewer postoperative complications, shorter intensive care unit stays, and faster recovery [8,10,13,14]. In the oncological context, this is particularly relevant, as early postoperative rehabilitation may enable timely initiation or continuation of systemic therapy, a cornerstone of multimodal treatment strategies in esophageal cancer. Robotic esophagectomy should therefore be viewed not merely as a technical refinement, but as a strategic approach to optimizing the completeness of multimodal oncological care.

Against this background, the present study aimed to characterize whether the institutional transition from open to hybrid and fully robotic Ivor Lewis esophagectomy was accompanied by an evolving profile of rare but clinically relevant complications. In a consecutive cohort of 407 patients, we demonstrate that full robotic RAMIE was associated with a markedly increased rate of chylothorax, whereas the incidence of neo-esophagus–airway fistula was significantly reduced compared with open surgery. Paraconduit herniation rates remained low within the 12-month observation period and did not differ significantly between approaches.

### 4.1. Patient Selection and Evolving Practice

The robotic cohorts in this study were characterized by significantly higher comorbidity burden, reflected by an increased Charlson Comorbidity Index and a higher prevalence of coronary artery disease, as well as by older patient age. These differences most likely represent an era effect driven by increasing institutional confidence in minimally invasive and robotic techniques, which gradually expanded the indication for curative surgery to patients who would previously have been considered high-risk candidates.

In parallel, the predominance of adenocarcinoma and the higher utilization of neoadjuvant therapy in the later cohorts reflect well-established epidemiological and treatment trends in Western esophageal cancer populations. While such baseline shifts are inherent to sequential cohort designs, adjustment for relevant covariates was performed to mitigate confounding. Nevertheless, the findings must be interpreted in the context of an evolving surgical program and continuous improvements in perioperative management over time.

### 4.2. Paraconduit Herniation

In contrast to previous reports describing paraconduit herniation rates of 5–15% after minimally invasive esophagectomy, with markedly increased odds compared to open procedures, the incidence observed in our cohort was low and did not differ significantly between approaches within 12 months. The most plausible explanation is the limited observation window, as published series report median times to diagnosis ranging from 8 to 24 months, and herniation is often a late-onset complication [16,30,31,32,33,34,35,36,37]. Importantly, when restricting analyses to 12-month survivors, herniation rates in HRB and FRB (3.6% each) exceeded those observed after OPE (0.9%), supporting the concept that reduced adhesion formation and a wider non-adherent hiatal gap may predispose to herniation after minimally invasive techniques. Despite the low absolute frequency, the clinical relevance of this complication is underscored by the fact that 6 of 8 affected patients required surgical repair. Given the limited number of events and the well-recognized late-onset nature of this complication, the regression model for paraconduit herniation is underpowered and the directional trend toward higher rates after minimally invasive approaches should be interpreted as hypothesis-generating—a conclusion consistent with the published literature [30,31,32,33,34,35] and one that warrants confirmation in larger cohorts with extended follow-up.

### 4.3. Chylothorax

The most striking finding of this study was the five-fold higher chylothorax rate after FRB (12.4%) compared with OPE and HRB (2.5–2.7%). Surgical approach remained the only independent predictor in multivariable analysis, suggesting that this phenomenon reflects a procedure-specific mechanism rather than patient-related risk factors alone. Notably, FRB was also associated with a significantly higher lymph node yield, supporting the interpretation that increased oncological radicality and extensive D2 lymphadenectomy contribute directly to the observed chylothorax burden [19,20].

However, our data also suggest that the underlying pathophysiology may differ from the classical paradigm of thoracic duct injury. In three FRB patients with persistent chylothorax who underwent radiographic lymphangiography, the thoracic duct was intact in all cases, and contrast leakage originated from the abdominal lymphadenectomy field near the celiac axis. The chylous fluid appeared to ascend transhiatally through a non-adherent hiatal gap into the thoracic cavity, where negative intrathoracic pressure and postoperative drainage suction may further promote lymphatic flow.

This observation supports a distinct mechanism of transhiatal lymphatic leakage rather than direct disruption of the thoracic duct. Importantly, this mechanistic concept is consistent with the operative technique: the hiatal opening was not routinely narrowed by cruroplasty, and the abdominal phase of FRB was performed with extensive D2 lymphadenectomy using predominantly monopolar dissection. In contrast, lymphatic structures during the abdominal phase in OPE and HRB were more frequently sealed using vessel-sealing devices. This technical difference may represent an additional contributing factor and warrants further evaluation, as standardized sealing strategies in robotic D2 lymphadenectomy could potentially reduce postoperative chylothorax rates.

Despite the increased incidence, all FRB-associated chylothorax cases were managed conservatively, and no increase in major complications or mortality was observed. In this context, the elevated chylothorax rate may represent an acceptable trade-off for enhanced lymphadenectomy and improved oncological staging, provided that awareness of this complication leads to early diagnosis and structured conservative management pathways.

### 4.4. Neo-Esophagus–Airway Fistula

Neo-esophagus–airway fistulas occurred approximately eight-fold more frequently after OPE than after FRB (4.9% vs. 0.6%). All fistula events were confined to patients who had received neoadjuvant therapy, and mortality in the open fistula subgroup was substantial: 7 of 8 affected patients died within 12 months, predominantly from recurrent sepsis and progressive respiratory failure—causes consistent with fistula-attributable mortality, as persistent airway communication inevitably perpetuates aspiration and chronic septic burden.

The pathophysiology of this complication is likely multifactorial. While anastomotic insufficiency may contribute as a cofactor, the markedly higher fistula rate after open surgery suggests that direct mechanical trauma to the posterior tracheobronchial wall during blunt thoracic dissection in a restricted operative field represents the primary underlying mechanism. In neoadjuvant-pretreated patients with therapy-induced tissue fragility, even microtrauma to the membranous trachea or left main bronchus may establish the conditions for subsequent fistula development. By contrast, robotic instrumentation enables stable exposure with reduced mechanical traction and precise, magnified dissection under tremor-filtered instrumentation—properties that may limit intraoperative airway wall injury independent of anastomotic healing [12].

Given the limited number of events, statistical certainty remains constrained; nevertheless, the magnitude of the observed effect suggests that robotic techniques confer a clinically meaningful protective advantage against this highly morbid complication. No mortality was attributable to chylothorax or paraconduit herniation within the observation period.

### 4.5. Anastomotic Leak and Perioperative Advantages

The comparatively high anastomotic leak rates across all cohorts require contextual interpretation. Since 2012, our institution has implemented routine postoperative day-5 endoscopy combined with prophylactic endoluminal vacuum therapy, thereby capturing even minor mucosal defects or early ischemic changes that may remain undetected under more restrictive diagnostic definitions. This approach likely increases reported leak rates while simultaneously enabling early therapeutic intervention, which may explain the absence of excess mortality despite high documented incidence.

Nevertheless, a stepwise reduction in leak rates from OPE to HRB and FRB was observed, consistent with the enhanced precision and reproducibility of robotic reconstruction. Beyond anastomotic outcomes, FRB demonstrated clear perioperative advantages, including reduced blood loss, shorter ICU and hospital stay, fewer pneumonias, and higher lymph node yield. Collectively, these findings align with the established benefits of minimally invasive surgery and highlight that robotic esophagectomy may improve postoperative recovery without compromising safety, even in increasingly comorbid patient populations [8,9,10].

### 4.6. Clinical Implications

From a clinical standpoint, our findings support the continued implementation of robotic Ivor Lewis esophagectomy in high-volume centers, even in older and comorbid patients, provided that structured perioperative pathways are in place. The observed increase in chylothorax after FRB should not be interpreted solely as a complication of technical failure, but rather as a predictable consequence of extensive abdominal lymphadenectomy and the presence of a non-adherent hiatal passage.

This has direct implications for postoperative management. Early recognition, prompt dietary modification (medium-chain triglyceride diet), and standardized drainage strategies appear appropriate first-line measures. In persistent cases, selective lymphangiography may be useful not only to exclude thoracic duct injury but also to identify abdominal transhiatal leakage patterns. From a preventive perspective, refinement of robotic D2 lymphadenectomy—particularly standardized sealing of lymphatic structures during the abdominal phase—may represent a relevant target for future investigation. Specifically, future studies should evaluate whether predominant monopolar dissection during robotic abdominal lymphadenectomy increases lymphatic leakage compared with vessel-sealing strategies, and whether a standardized sealing-based technique could reduce postoperative chylothorax rates.

Furthermore, the shared anatomical substrate of transhiatal chylous drainage and paraconduit herniation highlights the hiatus as a critical surgical interface. Long-term surveillance is therefore essential, as paraconduit herniation is likely underdetected within short follow-up intervals. Preventive strategies such as selective cruroplasty, hiatal fixation techniques, or standardized conduit positioning protocols warrant prospective evaluation, ideally within multicenter robotic programs.

### 4.7. Limitations

This study has several limitations inherent to its retrospective single-center design. All three surgical approaches were performed by a total of three senior attending surgeons. Open procedures were performed by one experienced esophageal surgeon, while robot-assisted procedures were performed by two surgeons within a structured dual-console training program to ensure technical consistency across all robotic cases. All critical operative steps—including mediastinal dissection, lymphadenectomy, and intrathoracic anastomosis—were performed exclusively by these attending surgeons; no complex steps were delegated to trainees. Importantly, the lead surgeon who introduced robotic techniques at our institution—first HRB, then FRB—was not an inexperienced operator: at the time of program initiation, he had already performed more than 100 robot-assisted procedures and had accumulated more than 10 years of experience in esophageal surgery. While surgeon and era effects cannot be fully isolated in a sequential cohort design, the pathophysiological nature of the primary endpoints and the multivariable confirmation of surgical approach as the sole independent predictor of chylothorax support a technique-driven rather than surgeon-driven interpretation of findings.

First, the sequential introduction of open, hybrid, and fully robotic techniques introduces an unavoidable era effect. Observed differences in baseline characteristics therefore reflect not only patient-related factors but also evolving institutional experience, expanding surgical indications, and changes in perioperative management over time. Although multivariable adjustment was performed, residual confounding cannot be excluded.

Second, the 12-month follow-up window was chosen to ensure standardized comparability across cohorts, but likely underestimates the true long-term incidence of paraconduit herniation, which is well recognized as a late-onset complication. Extended follow-up analyses are currently being conducted and will be reported separately. Third, our institution applies a deliberately sensitive definition of anastomotic leak—supported by routine postoperative day-5 endoscopy and prophylactic endoluminal vacuum therapy—which captures subclinical anastomotic abnormalities that would remain undetected under more restrictive diagnostic criteria, limiting direct comparability with other published series.

Fourth, event numbers for paraconduit herniation and neo-esophagus–airway fistula were low, resulting in limited statistical power and wide confidence intervals, particularly in multivariable models. Patient-reported outcomes and formal quality-of-life assessments were not available and should be addressed in future prospective studies. Fifth, the observed reductions in ICU and hospital stay cannot be attributed exclusively to the surgical approach. A mid-2018 institutional reorganization—transitioning from a surgery-dedicated ICU with extended monitoring protocols to a centralized interdisciplinary structure under anesthesiology management, with earlier step-down criteria for clinically stable patients—coincided temporally with the introduction of robotic surgery and represents an independent era effect that cannot be fully disentangled from technique-specific benefits in this retrospective design. Longer follow-up and multicenter validation will be essential to confirm the generalizability of the observed complication profile.

## 5. Conclusions

Robotic Ivor Lewis esophagectomy is associated with a distinct complication profile compared to open and hybrid approaches. Fully robotic surgery demonstrated a significantly higher chylothorax rate, attributable to the enhanced radicality of transhiatal celiac lymphadenectomy enabled by the robotic platform; however, conservative management was sufficient in all cases and no chylothorax-related mortality was observed. Neo-esophagus–airway fistula occurred approximately eight-fold more frequently after open esophagectomy than after fully robotic surgery, with all events confined to neoadjuvant-treated patients, supporting a technique-specific mechanism related to intraoperative tracheobronchial trauma. Paraconduit herniation rates did not differ significantly within the 12-month observation window, though the directional trend toward higher rates after minimally invasive approaches warrants confirmation in larger cohorts with extended follow-up. Taken together, these findings demonstrate that fully robotic esophagectomy can be implemented safely in a high-volume center, with a complication profile that differs qualitatively—rather than quantitatively—from open surgery, and with meaningful reductions in the most clinically devastating complications.

## Figures and Tables

**Table 1:** Baseline characteristics of the study cohort.

Variable	OPE (*n* = 163)	HRB (*n* = 75)	FRB (*n* = 169)	*p*
**Demographics**				
Age, years—median (IQR)	62.3 (55.5–68.5)	63.6 (59.0–72.4)	66.0 (59.4–71.1)	0.012
Male sex—*n* (%)	130 (79.8%)	62 (82.7%)	143 (84.6%)	0.508
BMI, kg/m^2^—median (IQR)	25.3 (22.5–29.0)	26.2 (24.1–31.2)	25.5 (23.1–28.4)	0.081
**Comorbidities**				
ASA classification—*n* (%)				0.153
ASA II	97 (59.5%)	40 (53.3%)	83 (49.1%)	
ASA III^+^	59 (36.2%)	33 (44.0%)	82 (48.5%)	
Charlson Comorbidity Index—median (IQR)	4.0 (3.0–5.0)	5.0 (4.0–6.0)	5.0 (4.0–6.0)	<0.001
Diabetes mellitus—*n* (%)	21 (12.9%)	19 (25.3%)	32 (18.9%)	0.056
Coronary artery disease—*n* (%)	12 (7.4%)	12 (16.0%)	33 (19.5%)	0.005
COPD—*n* (%)	16 (9.8%)	4 (5.3%)	12 (7.1%)	0.437
**Tumor characteristics**				
Histology—*n* (%)				<0.001
Adenocarcinoma	102 (62.6%)	67 (89.3%)	143 (84.6%)	
Squamous cell carcinoma	61 (37.4%)	8 (10.7%)	26 (15.4%)	
cT3/T4—*n* (%)	99 (60.7%)	47 (62.7%)	112 (66.3%)	0.711
cN^+^—*n* (%)	134 (82.2%)	54 (72.0%)	120 (71.0%)	0.042
**Neoadjuvant therapy**				
Any neoadjuvant therapy—*n* (%)	129 (79.1%)	60 (80.0%)	155 (91.7%)	0.003
None	34 (20.9%)	15 (20.0%)	14 (8.3%)	
Chemotherapy (FLOT)	39 (23.9%)	25 (33.3%)	56 (33.1%)	
Radiochemotherapy (CROSS)	90 (55.2%)	35 (46.7%)	97 (57.4%)	

Notes: Continuous variables are presented as median (IQR) and compared using the Kruskal–Wallis test. Categorical variables are presented as *n* (%) and compared using Pearson’s chi-square test or Fisher’s exact test where appropriate. Abbreviations: OPE, open esophagectomy; HRB, hybrid robot-assisted minimally invasive esophagectomy; FRB, full robotic robot-assisted minimally invasive esophagectomy; BMI, body mass index; ASA, American Society of Anesthesiologists; CCI, Charlson Comorbidity Index; FLOT, fluorouracil/leucovorin/oxaliplatin/docetaxel; CROSS, carboplatin/paclitaxel with concurrent radiotherapy.

**Table 2:** Primary and secondary outcomes.

Outcome	OPE (*n* = 163)	HRB (*n* = 75)	FRB (*n* = 169)	*p*
**One-year survival and primary endpoints (within 12 months postoperatively)**				
1-year overall survival—*n* (%)	117/163 (71.8%)	56/75 (74.7%)	139/169 (82.2%)	0.073
Paraconduit Herniation—*n* (%)	1/163 (0.6%)	2/75 (2.7%)	5/169 (3.0%)	0.272
—in 1-year survivors	1/117 (0.9%)	2/56 (3.6%)	5/139 (3.6%)	—
Chylothorax—*n* (%)	4/163 (2.5%)	2/75 (2.7%)	21/169 (12.4%)	<0.001
—in 1-year survivors	4/117 (3.4%)	2/56 (3.6%)	16/139 (11.5%)	—
Neo-Esophagus-Airway Fistula—*n* (%)	8/163 (4.9%)	1/75 (1.3%)	1/169 (0.6%)	0.031
—in 1-year survivors	1/117 (0.9%)	1/56 (1.8%)	0/139 (0.0%)	—
**Secondary endpoints—postoperative complications**				
Anastomotic leak—*n* (%)	61 (37.4%)	17 (22.7%)	23 (13.6%)	<0.001
Pneumonia—*n* (%)	47 (28.8%)	18 (24.0%)	22 (13.0%)	0.002
Clavien–Dindo ≥ IIIb—*n* (%)	35 (21.5%)	11 (14.7%)	22 (13.0%)	0.106
30-day mortality—*n* (%)	3 (1.8%)	0 (0.0%)	3 (1.8%)	0.502
In-hospital mortality—*n* (%)	14 (8.6%)	4 (5.3%)	5 (3.0%)	0.084
**Secondary endpoints—perioperative metrics**				
Operative time, min—median (IQR)	306 (274–357)	495 (418–598)	415 (366–488)	<0.001
Blood loss, mL—median (IQR)	400 (0–600)	0 (0–200)	80 (50–200)	<0.001
ICU stay, days—median (IQR)	10.0 (7.0–18.5)	3.0 (2.0–7.0)	2.0 (1.0–3.0)	<0.001
Hospital stay, days—median (IQR)	22.6 (16.0–46.1)	20.0 (13.0–28.0)	16.0 (13.0–23.2)	<0.001
Lymph nodes harvested—median (IQR)	25 (19–32)	29 (23–34)	32 (27–38)	<0.001

Notes: Primary endpoints were assessed within 12 months postoperatively. Rates are additionally reported among 12-month survivors to account for competing risk of early mortality. Abbreviations: OPE, open esophagectomy; HRB, hybrid robot-assisted minimally invasive esophagectomy; FRB, full robotic robot-assisted minimally invasive esophagectomy; ICU, intensive care unit; EVT, endoluminal vacuum therapy.

**Table 3:** Logistic regression analysis of primary endpoints.

	Univariable	Univariable	Univariable	Multivariable	Multivariable	Multivariable
Variable	OR	95% CI	*p*	OR	95% CI	*p*
**(A) Paraconduit Herniation (*n* = 407; events = 8)**						
OPE vs. FRB	0.20	0.02–1.75	0.147	0.15	0.01–1.50	0.106
HRB vs. FRB	0.90	0.17–4.74	0.900	1.07	0.19–6.07	0.938
Age (per year)	—	—	—	0.93	0.88–0.99	0.019
Sex (male)	—	—	—	0.32	0.06–1.61	0.168
BMI	—	—	—	1.01	0.88–1.15	0.903
ASA III/IV	—	—	—	0.69	0.15–3.25	0.641
FLOT chemotherapy	—	—	—	1.74	0.04–67.5	0.767
CROSS radiochemotherapy	—	—	—	3.70	0.12–117	0.458
**(B) Chylothorax (*n* = 407; events = 27)**						
OPE vs. FRB	0.18	0.06–0.53	0.002	0.16	0.05–0.48	0.001
HRB vs. FRB	0.19	0.04–0.85	0.029	0.17	0.04–0.78	0.022
Age (per year)	—	—	—	0.98	0.95–1.02	0.439
Sex (male)	—	—	—	0.98	0.33–2.94	0.976
BMI	—	—	—	1.01	0.94–1.10	0.738
ASA III/IV	—	—	—	0.78	0.35–1.77	0.558
FLOT chemotherapy	—	—	—	0.87	0.23–3.36	0.839
CROSS radiochemotherapy	—	—	—	0.76	0.21–2.73	0.671
**(C) Neo-Esophagus-Airway Fistula (*n* = 407; events = 10)**						
OPE vs. FRB	8.67	1.07–70.1	0.043	4.84	0.87–26.9	0.072
HRB vs. FRB	2.27	0.14–36.8	0.564	1.73	0.14–21.3	0.668
Age (per year)	—	—	—	0.94	0.89–1.00	0.041
Sex (male)	—	—	—	2.30	0.33–16.1	0.401
BMI	—	—	—	0.90	0.78–1.04	0.155
ASA III/IV	—	—	—	0.74	0.19–2.87	0.658
Any neoadjuvant therapy †	—	—	—	6.46	0.24–176	0.269

Notes: Odds ratios (OR) are presented with 95% confidence intervals (CI). FRB served as the reference category. Multivariable models were adjusted for age, sex, BMI, ASA classification, and neoadjuvant therapy. Abbreviations: OR, odds ratio; CI, confidence interval; OPE, open esophagectomy; HRB, hybrid robot-assisted minimally invasive esophagectomy; FRB, full robotic robot-assisted minimally invasive esophagectomy; BMI, body mass index; ASA, American Society of Anesthesiologists; chemotherapy or radiochemotherapy †.

## Data Availability

The data supporting the findings of this study are not publicly available due to privacy and ethical restrictions.
